# Methyl 4-(3-chloro­prop­oxy)-3-methoxy­benzoate

**DOI:** 10.1107/S1600536808043274

**Published:** 2009-01-08

**Authors:** Min Zhang, Ran-Zhe Lu, Lu-Na Han, Bin Wang, Hai-Bo Wang

**Affiliations:** a College of Science, Nanjing University of Technology, Xinmofan Road No.5 Nanjing, Nanjing 210009, People’s Republic of China

## Abstract

In the title compound, C_12_H_15_ClO_4_, the molecules are linked by C—H⋯O interactions.

## Related literature

For general background, see: Knesl *et al.* (2006 ▶). For bond-length data, see: Allen *et al.* (1987 ▶).
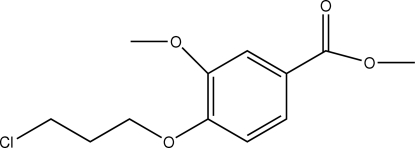


## Experimental

### 

#### Crystal data

C_12_H_15_ClO_4_
*M* *_r_* = 258.69
Monoclinic, 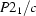

*a* = 8.4980 (17) Å
*b* = 17.349 (4) Å
*c* = 8.8440 (18) Å
β = 106.46 (3)°
*V* = 1250.5 (5) Å^3^
*Z* = 4
Mo *K*α radiation
μ = 0.31 mm^−1^
*T* = 294 (2) K
0.30 × 0.20 × 0.10 mm

#### Data collection

Enraf–Nonius CAD-4 diffractometer
Absorption correction: ψ scan (North *et al.*, 1968 ▶) *T*_min_ = 0.914, *T*_max_ = 0.970
2431 measured reflections
2274 independent reflections
1575 reflections with *I* > 2σ(*I*)
*R*_int_ = 0.048
3 standard reflections frequency: 120 min intensity decay: 1%

#### Refinement

*R*[*F* ^2^ > 2σ(*F* ^2^)] = 0.068
*wR*(*F* ^2^) = 0.176
*S* = 1.01
2274 reflections
154 parameters
H-atom parameters constrained
Δρ_max_ = 0.35 e Å^−3^
Δρ_min_ = −0.29 e Å^−3^

### 

Data collection: *CAD-4 Software* (Enraf–Nonius, 1989 ▶); cell refinement: *CAD-4 Software*; data reduction: *XCAD4* (Harms & Wocadlo, 1995 ▶); program(s) used to solve structure: *SHELXS97* (Sheldrick, 2008 ▶); program(s) used to refine structure: *SHELXL97* (Sheldrick, 2008 ▶); molecular graphics: *ORTEP-3 for Windows* (Farrugia, 1997 ▶) and *PLATON* (Spek, 2003 ▶); software used to prepare material for publication: *SHELXTL* (Sheldrick, 2008 ▶).

## Supplementary Material

Crystal structure: contains datablocks D, I. DOI: 10.1107/S1600536808043274/hk2602sup1.cif

Structure factors: contains datablocks I. DOI: 10.1107/S1600536808043274/hk2602Isup2.hkl

Additional supplementary materials:  crystallographic information; 3D view; checkCIF report

## Figures and Tables

**Table 1:** Hydrogen-bond geometry (Å, °)

*D*—H⋯*A*	*D*—H	H⋯*A*	*D*⋯*A*	*D*—H⋯*A*
C1—H1*B*⋯O2^i^	0.97	2.56	3.429 (6)	149
C2—H2*A*⋯O3^ii^	0.97	2.41	3.358 (6)	164

Symmetry codes: (i) 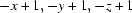; (ii) 
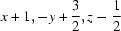
.

**Fig. 1.**
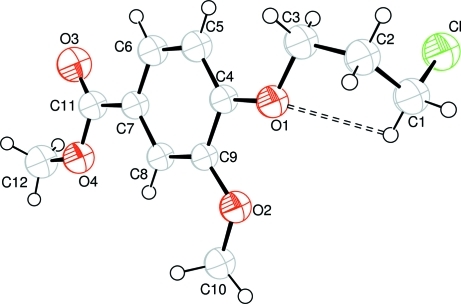
The molecular structure of the title molecule, with the atom-numbering scheme. Hydrogen bond is shown as dashed line.

**Fig. 2.**
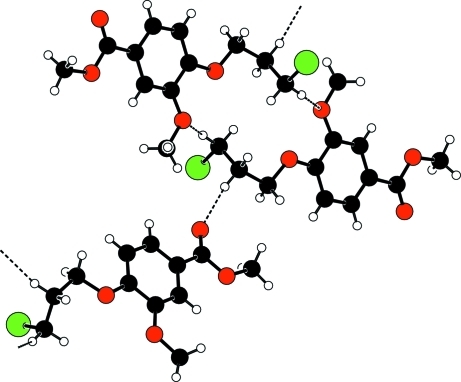
A partial packing diagram of the title compound. Hydrogen bonds are shown as dashed lines.

## supplementary crystallographic information

### Comment

As part of our ongoing studies on quinazoline derivatives (Knesl *et al.*, 2006), we report herein the crystal structure of the title compound.

In the molecule of the title compound (Fig 1), the bond lengths (Allen *et al.*,  1987) and angles are within normal ranges. Ring A (C4-C9) is, of course, planar. The intramolecular C-H···O hydrogen bond (Table 1) results in the formation of a five-membered ring B (O1/C1-C3/H1A), having envelope conformation with C2 atom displaced by -0.668 (3) Å from the plane of the other ring atoms.

In the crystal structure, intermolecular C-H···O hydrogen bonds (Table 1) link the molecules (Fig. 2), in which they may be effective in the stabilization of the structure.

### Experimental

For the preparation of the title compound, methyl 3-methoxy-4-hydroxybenzoate (55 mmol), 1-bromo-3-chloropropane (165 mmol) and potassium carbonate (275 mmol) were mixed with DMF (60 ml), and then the mixture was heated to reflux for 2 h. Reaction progress was monitored by TLC. After cooling and filtration, the title compound was obtained (yield; 93.7%, m.p. 384 K). Crystals suitable for X-ray analysis were obtained by slow evaporation of an ethyl acetate solution.

### Refinement

H atoms were positioned geometrically, with C-H = 0.93, 0.97 and 0.96 Å for aromatic, methylene and methyl H, respectively, and constrained to ride on their parent atoms, with U_iso_(H) = xU_eq_(C), where x = 1.5 for methyl H and x = 1.2 for all other H atoms.

### Crystal data

**Table:**

C_12_H_15_ClO_4_	*F*(000) = 544
*M**_r_* = 258.69	*D*_x_ = 1.374 Mg m^−^^3^
Monoclinic, *P*2_1_/*c*	Melting point: 384 K
Hall symbol: -P 2ybc	Mo *K*α radiation, λ = 0.71073 Å
*a* = 8.4980 (17) Å	Cell parameters from 25 reflections
*b* = 17.349 (4) Å	θ = 10–13°
*c* = 8.8440 (18) Å	µ = 0.31 mm^−^^1^
β = 106.46 (3)°	*T* = 294 K
*V* = 1250.5 (5) Å^3^	Block, colorless
*Z* = 4	0.30 × 0.20 × 0.10 mm

### Data collection

**Table:**

Enraf–Nonius CAD-4 diffractometer	1575 reflections with *I* > 2σ(*I*)
Radiation source: fine-focus sealed tube	*R*_int_ = 0.048
graphite	θ_max_ = 25.3°, θ_min_ = 2.4°
ω/2θ scans	*h* = 0→10
Absorption correction: ψ scan (North *et al.*, 1968)	*k* = 0→20
*T*_min_ = 0.914, *T*_max_ = 0.970	*l* = −10→10
2431 measured reflections	3 standard reflections every 120 min
2274 independent reflections	intensity decay: 1%

### Refinement

**Table:**

Refinement on *F*^2^	Primary atom site location: structure-invariant direct methods
Least-squares matrix: full	Secondary atom site location: difference Fourier map
*R*[*F*^2^ > 2σ(*F*^2^)] = 0.068	Hydrogen site location: inferred from neighbouring sites
*wR*(*F*^2^) = 0.176	H-atom parameters constrained
*S* = 1.01	*w* = 1/[σ^2^(*F*_o_^2^) + (0.050*P*)^2^ + 3.3*P*] where *P* = (*F*_o_^2^ + 2*F*_c_^2^)/3
2274 reflections	(Δ/σ)_max_ < 0.001
154 parameters	Δρ_max_ = 0.35 e Å^−^^3^
0 restraints	Δρ_min_ = −0.29 e Å^−^^3^

### Special details

**Table:**

Geometry. All esds (except the esd in the dihedral angle between two l.s. planes) are estimated using the full covariance matrix. The cell esds are taken into account individually in the estimation of esds in distances, angles and torsion angles; correlations between esds in cell parameters are only used when they are defined by crystal symmetry. An approximate (isotropic) treatment of cell esds is used for estimating esds involving l.s. planes.
Refinement. Refinement of F^2^ against ALL reflections. The weighted R-factor wR and goodness of fit S are based on F^2^, conventional R-factors R are based on F, with F set to zero for negative F^2^. The threshold expression of F^2^ > 2sigma(F^2^) is used only for calculating R-factors(gt) etc. and is not relevant to the choice of reflections for refinement. R-factors based on F^2^ are statistically about twice as large as those based on F, and R- factors based on ALL data will be even larger.

### Fractional atomic coordinates and isotropic or equivalent isotropic displacement parameters (Å^2^)

**Table:**

	*x*	*y*	*z*	*U*_iso_*/*U*_eq_	
Cl	0.27901 (16)	0.58276 (8)	0.10729 (14)	0.0679 (4)	
O1	0.3056 (3)	0.60776 (15)	0.5453 (3)	0.0480 (7)	
O2	0.1798 (3)	0.50211 (15)	0.6779 (3)	0.0491 (7)	
O3	−0.2908 (4)	0.74808 (19)	0.7610 (4)	0.0711 (10)	
O4	−0.2715 (3)	0.62804 (17)	0.8531 (4)	0.0578 (8)	
C1	0.4233 (6)	0.5519 (3)	0.2872 (5)	0.0580 (12)	
H1A	0.3693	0.5170	0.3424	0.070*	
H1B	0.5119	0.5239	0.2627	0.070*	
C2	0.4929 (5)	0.6187 (3)	0.3921 (5)	0.0554 (11)	
H2A	0.5409	0.6546	0.3337	0.066*	
H2B	0.5803	0.6000	0.4807	0.066*	
C3	0.3697 (5)	0.6615 (2)	0.4548 (5)	0.0532 (11)	
H3A	0.4223	0.7043	0.5207	0.064*	
H3B	0.2819	0.6817	0.3684	0.064*	
C4	0.1809 (5)	0.6315 (2)	0.6028 (4)	0.0401 (9)	
C5	0.1190 (5)	0.7053 (2)	0.5911 (5)	0.0488 (10)	
H5A	0.1656	0.7438	0.5444	0.059*	
C6	−0.0123 (5)	0.7224 (2)	0.6489 (5)	0.0489 (10)	
H6A	−0.0547	0.7722	0.6390	0.059*	
C7	−0.0814 (5)	0.6657 (2)	0.7216 (4)	0.0424 (9)	
C8	−0.0174 (5)	0.5915 (2)	0.7335 (4)	0.0404 (9)	
H8A	−0.0638	0.5533	0.7811	0.048*	
C9	0.1129 (4)	0.5731 (2)	0.6768 (4)	0.0378 (8)	
C10	0.1078 (5)	0.4411 (2)	0.7422 (5)	0.0495 (10)	
H10A	0.1631	0.3937	0.7342	0.074*	
H10B	−0.0061	0.4366	0.6849	0.074*	
H10C	0.1176	0.4517	0.8511	0.074*	
C11	−0.2236 (5)	0.6865 (2)	0.7781 (5)	0.0478 (10)	
C12	−0.4095 (6)	0.6411 (3)	0.9117 (6)	0.0670 (14)	
H12A	−0.4308	0.5957	0.9646	0.101*	
H12B	−0.5039	0.6531	0.8255	0.101*	
H12C	−0.3865	0.6835	0.9846	0.101*	

### Atomic displacement parameters (Å^2^)

**Table:**

	*U*^11^	*U*^22^	*U*^33^	*U*^12^	*U*^13^	*U*^23^
Cl	0.0710 (8)	0.0750 (8)	0.0547 (7)	0.0031 (6)	0.0130 (6)	0.0007 (6)
O1	0.0477 (16)	0.0459 (16)	0.0542 (17)	−0.0020 (12)	0.0206 (13)	0.0082 (13)
O2	0.0481 (16)	0.0364 (15)	0.0657 (19)	0.0022 (12)	0.0211 (14)	0.0077 (13)
O3	0.079 (2)	0.057 (2)	0.086 (2)	0.0270 (17)	0.037 (2)	0.0083 (18)
O4	0.0487 (17)	0.0563 (19)	0.074 (2)	0.0088 (14)	0.0267 (16)	−0.0028 (16)
C1	0.060 (3)	0.055 (3)	0.062 (3)	0.006 (2)	0.022 (2)	0.006 (2)
C2	0.048 (2)	0.063 (3)	0.057 (3)	−0.003 (2)	0.018 (2)	0.008 (2)
C3	0.059 (3)	0.044 (2)	0.054 (3)	−0.009 (2)	0.012 (2)	0.004 (2)
C4	0.041 (2)	0.043 (2)	0.036 (2)	−0.0010 (17)	0.0103 (16)	−0.0003 (16)
C5	0.063 (3)	0.034 (2)	0.050 (2)	−0.0083 (19)	0.016 (2)	−0.0017 (18)
C6	0.056 (3)	0.035 (2)	0.051 (2)	0.0081 (18)	0.006 (2)	−0.0028 (18)
C7	0.043 (2)	0.042 (2)	0.041 (2)	0.0039 (17)	0.0092 (17)	−0.0063 (17)
C8	0.039 (2)	0.040 (2)	0.040 (2)	−0.0037 (16)	0.0073 (16)	0.0028 (17)
C9	0.041 (2)	0.0330 (19)	0.040 (2)	0.0027 (16)	0.0114 (16)	−0.0013 (16)
C10	0.057 (3)	0.034 (2)	0.062 (3)	0.0011 (18)	0.023 (2)	0.0053 (19)
C11	0.054 (2)	0.042 (2)	0.043 (2)	0.0044 (19)	0.0058 (19)	−0.0037 (18)
C12	0.052 (3)	0.083 (4)	0.073 (3)	0.007 (2)	0.030 (2)	−0.014 (3)

### Geometric parameters (Å, °)

**Table:**

Cl—C1	1.794 (5)	C4—C5	1.378 (5)
O1—C3	1.433 (5)	C4—C9	1.415 (5)
O1—C4	1.362 (4)	C5—C6	1.385 (6)
O2—C9	1.355 (4)	C5—H5A	0.9300
O2—C10	1.420 (4)	C6—C7	1.394 (6)
O3—C11	1.201 (5)	C6—H6A	0.9300
O4—C11	1.336 (5)	C7—C8	1.390 (5)
O4—C12	1.429 (5)	C7—C11	1.478 (6)
C1—C2	1.498 (6)	C8—C9	1.377 (5)
C1—H1A	0.9700	C8—H8A	0.9300
C1—H1B	0.9700	C10—H10A	0.9600
C2—C3	1.512 (6)	C10—H10B	0.9600
C2—H2A	0.9700	C10—H10C	0.9600
C2—H2B	0.9700	C12—H12A	0.9600
C3—H3A	0.9700	C12—H12B	0.9600
C3—H3B	0.9700	C12—H12C	0.9600
			
C4—O1—C3	118.1 (3)	C5—C6—C7	120.5 (4)
C9—O2—C10	116.9 (3)	C5—C6—H6A	119.8
C11—O4—C12	117.1 (3)	C7—C6—H6A	119.8
Cl—C1—H1A	109.3	C8—C7—C6	118.9 (4)
Cl—C1—H1B	109.3	C8—C7—C11	122.7 (4)
C2—C1—Cl	111.6 (3)	C6—C7—C11	118.4 (4)
C2—C1—H1A	109.3	C9—C8—C7	121.6 (4)
C2—C1—H1B	109.3	C9—C8—H8A	119.2
H1A—C1—H1B	108.0	C7—C8—H8A	119.2
C1—C2—C3	114.5 (4)	O2—C9—C8	125.9 (3)
C1—C2—H2A	108.6	O2—C9—C4	115.4 (3)
C1—C2—H2B	108.6	C8—C9—C4	118.6 (3)
C3—C2—H2A	108.6	O2—C10—H10A	109.5
C3—C2—H2B	108.6	O2—C10—H10B	109.5
H2A—C2—H2B	107.6	H10A—C10—H10B	109.5
O1—C3—C2	107.3 (3)	O2—C10—H10C	109.5
O1—C3—H3A	110.3	H10A—C10—H10C	109.5
O1—C3—H3B	110.3	H10B—C10—H10C	109.5
C2—C3—H3A	110.3	O3—C11—O4	122.5 (4)
C2—C3—H3B	110.3	O3—C11—C7	125.4 (4)
H3A—C3—H3B	108.5	O4—C11—C7	112.1 (3)
O1—C4—C5	125.0 (3)	O4—C12—H12A	109.5
O1—C4—C9	114.8 (3)	O4—C12—H12B	109.5
C5—C4—C9	120.2 (4)	H12A—C12—H12B	109.5
C4—C5—C6	120.2 (4)	O4—C12—H12C	109.5
C4—C5—H5A	119.9	H12A—C12—H12C	109.5
C6—C5—H5A	119.9	H12B—C12—H12C	109.5
			
Cl—C1—C2—C3	65.9 (4)	C10—O2—C9—C4	−176.1 (3)
C4—O1—C3—C2	−174.3 (3)	C7—C8—C9—O2	−177.5 (4)
C1—C2—C3—O1	60.5 (5)	C7—C8—C9—C4	−0.9 (6)
C3—O1—C4—C5	−5.2 (6)	O1—C4—C9—O2	−0.9 (5)
C3—O1—C4—C9	173.8 (3)	C5—C4—C9—O2	178.2 (4)
O1—C4—C5—C6	177.6 (4)	O1—C4—C9—C8	−177.9 (3)
C9—C4—C5—C6	−1.3 (6)	C5—C4—C9—C8	1.2 (6)
C4—C5—C6—C7	1.1 (6)	C12—O4—C11—O3	0.8 (6)
C5—C6—C7—C8	−0.7 (6)	C12—O4—C11—C7	−179.4 (3)
C5—C6—C7—C11	−178.7 (4)	C8—C7—C11—O3	−174.5 (4)
C6—C7—C8—C9	0.6 (6)	C6—C7—C11—O3	3.4 (6)
C11—C7—C8—C9	178.5 (4)	C8—C7—C11—O4	5.6 (5)
C10—O2—C9—C8	0.6 (6)	C6—C7—C11—O4	−176.5 (4)

### Hydrogen-bond geometry (Å, °)

**Table:**

*D*—H···*A*	*D*—H	H···*A*	*D*···*A*	*D*—H···*A*
C1—H1A···O1	0.97	2.56	2.906 (5)	101
C1—H1B···O2^i^	0.97	2.56	3.429 (6)	149
C2—H2A···O3^ii^	0.97	2.41	3.358 (6)	164

Symmetry codes: (i) −*x*+1, −*y*+1, −*z*+1; (ii) *x*+1, −*y*+3/2, *z*−1/2.
